# Monograph on Glycerin and Its Uses

**Published:** 1866-02

**Authors:** Henry Hartshorne

**Affiliations:** Member of the American Philosophical Society, &c.


A MONOGRAPH ON GLYCERIN AND ITS USES. By HENRY HARTSHORNE, M. D., Member of the American Philosophical Society, &c. Philadelphia: J. P. Lippincott & Co. 1865.

IN this neat little book of sixty-eight pages the compiler has given us nearly everything yet ascertained relative to the properties, chemical relations, medical and pharmaceutical uses, and physiological action of one of the most remarkable and important substances in our materia medica. Possessed of wonderful powers as a solvent and preservative, and at the same time almost as inert as water, its chief value in our art lies in its employment as a vehicle for the administration and application of drugs in a more unchangeable and concentrated form than those hitherto used. Internally, it has been somewhat employed as a medicine in cough mixtures, phthisis and other diseases, but its therapeutic or nutritive powers must be very slight. We think the author has also dwelt too much upon its importance in cutaneous diseases, and has attributed effects to the vehicle which belong entirely to the substances combined with it. As a substitute for fat in ointments, and for water or alcohol in washes, there can be no doubt, of its value in some cases, but by itself it has no curative action upon the skin. We can recommend this volume to our readers as an important chapter hitherto wanting in their works on materia medica.

